# Canine Olfactory Detection of SARS-COV2-Infected Patients: A One Health Approach

**DOI:** 10.3389/fpubh.2021.647903

**Published:** 2021-10-21

**Authors:** Rita de Cássia Carvalho Maia, Leucio Câmara Alves, Jeine Emanuele Santos da Silva, François Rémi Czyba, Jorge Antonio Pereira, Vincent Soistier, Clothilde Lecoq Julien, Dominique Grandjean, Anísio Francisco Soares

**Affiliations:** ^1^Veterinary Medicine Department, Federal Rural University of Pernambuco, Recife, Brazil; ^2^Animal Morphology and Physiology Department, Federal Rural University of Pernambuco, Recife, Brazil; ^3^Amarante do Brasil, Avenida Erasmo Braga, Rio de Janeiro, Brazil; ^4^National Veterinary School of Alfort, Maisons Alfort, France

**Keywords:** dog, COVID-19, odor, axillar, diagnosis, Latin America, one health

## Abstract

The aim of the present study is to apply the canine olfactory sensitivity to detect COVID-19-positive axillary sweat samples as a One Health approach in Latin America. One hundred volunteers with COVID-like symptoms were invited to participate, and both axillary sweat samples for dog detection and nasopharynx/oropharynx swabs for qPCR were collected. Two dogs, previously trained, detected 97.4% of the samples positive for COVID-19, including a false-negative qPCR-test, and the positive predictive value was 100% and the negative predictive value was 98.2%. Therefore, we can conclude that canine olfactory sensitivity can detect a person infected with COVID-19 through axillary sweat successfully and could be used as an alternative to screen them without invasive testing.

## Introduction

Recent events during the SARS-CoV2 Pandemic development, initiated in the Wuhan Province in China in November 2019 (1), have brought enormous challenges to a population adapted to the globalized aspects of daily life, contributing largely to the continuity of the disease development, and augmenting the Public Health impact (2, 3). Letting go of these connections has been difficult. On the other hand, the reach of current technologies combined with mutual scientific collaboration worldwide has brought faster and more efficient responses to health problems when associated with a stronger and broader view of One Health, especially considering the involvement of animals at different levels of the epidemiologic chain of the disease (4). Among the problems envisioned, we observed the need to lower the costs of diagnostic tests, since the actual diagnostic performance depends on testing during different stages of the disease directly, depending on viral load, or indirectly at later stages, depending on antibody production. The reduction of testing also minimizes environmental impacts due to the large use of disposable materials, mainly plastic. Moreover, these tests bring a large margin of error due to the false-negative results given the failure to detect viral load or antibody production and thereby, adding the burden of asymptomatic patients being left untested (5), propagating the infection freely and risking further human, animal, and environmental dissemination. Another major obstacle is the cost of testing a large number of people (6) especially for those in developing countries. Above all remains the logistics of reopening and returning from quarantines that will resume physical contact, and the invasiveness of current tests that depend on tracheal or blood collections.

Using canine smell to locate buried people, drugs, and ammunition in different environments is a well-known, recognized, and applied activity (7, 8). The reason for this is confidence in the canine response to the most diverse volatile compounds emitted. Evaluating the problem in a broader One Health approach like using dogs to detect chronic degenerative and proliferative diseases by identifying Volatile Organic Compounds (VOCs) produced in the metabolism in the body of a patient during the disease has been a practice used for many diseases, such as several types of cancer, Parkinson's disease, and viral diseases, including coronavirus disease-2019 (COVID-19) (9–16). The olfactory detection capacity of the dog allies the large nasal cavity with an expressive olfactory epithelium surface area containing 30% more olfactory receptors than those in humans (17). Additionally, during the active sniffing process, characterized by short and sharp inhalations, the air inhaled into the nostrils is directed to the dorsal nasal cavity, flowing back to the ethmoturbinates. This process allows a longer exposure time of VOCs to the olfactory chemoreceptors, with a continuous stimulation of the olfactory centers throughout the respiratory cycle (18). Taken together, the advantages to humans, the environment, and animals in this context, the present project has as its main objective the application of a new approach for early detection of COVID-19 in humans through canine olfactory sensitivity to counteract the many challenges imposed by this disease.

## Materials and Methods

**Figure 1:**
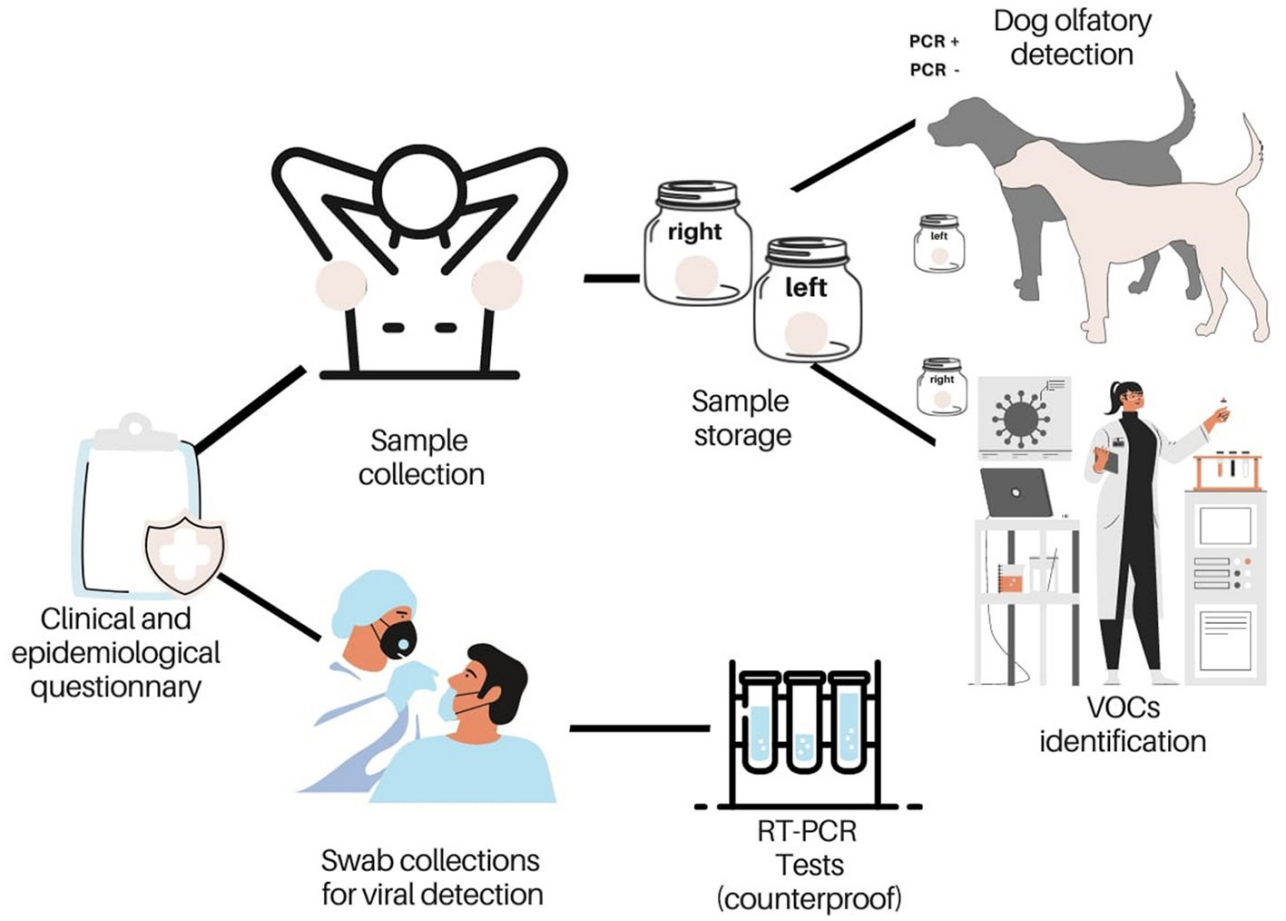
Schematic representation depicting sample collection.

One hundred volunteers were selected to participate in the research. During a visit to patients with flu syndrome symptoms by the team of health agents from the Municipality of Paudalho, the northern region of Recife, Brazil, they were invited to participate in the research and signed the Free and Informed Consent Form. They were instructed to remain without bathing or using perfumes for the next 24 h, and sample collection was carried out the next day. Two types of samples were collected, one for the olfactory test of the dogs and another for confirming the patient diagnosis which was also used as a counterproof of the result achieved by the dogs. A questionnaire of clinical and epidemiological interest was also applied. The sample collection for dog training was performed by asking the volunteer to place a cotton ball under each armpit for 20 min (Figure 1). The cotton pads were collected and placed separately in hermetically sealed glass jars and labeled to precisely identify the origin of the sample. Sample collections for viral detection by RT-PCR were performed using a combination of two rayon swabs from the nasopharynx and oropharynx, stored in a 0.85% saline buffer per volunteer. The samples were collected by professionally trained health professionals at the Paudalho Municipal Laboratory following all Biosafety rules. After collection, the material was packed and stored in a cooled thermal box to avoid contamination of the samples during transportation. They were delivered for analysis within 2 h of collection.

**Figure 2:**
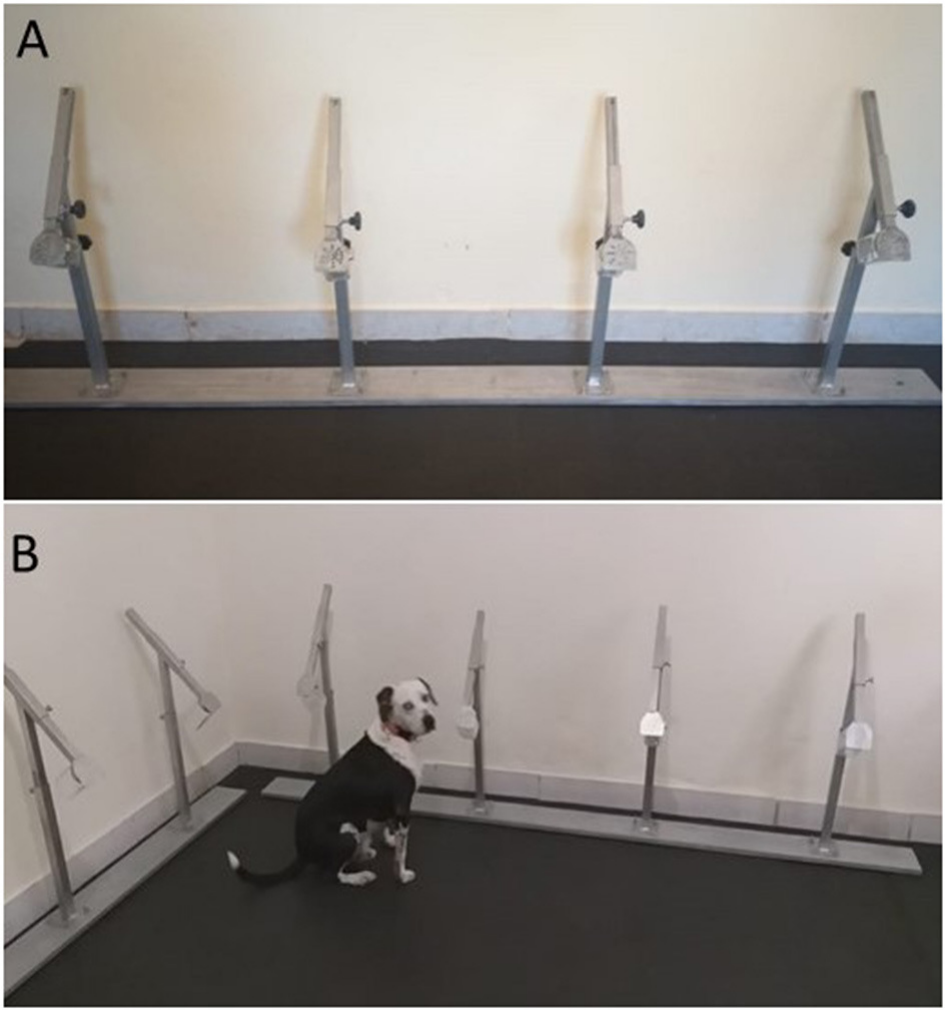
The experiment of olfactory detection by dogs. **(A)** Testing line of samples. **(B)** Male dog, Sinatra, indicating the positive sample by sitting in front of the sample.

The cotton balls samples were stored separately, one was used for the detection of odors by dogs, and the other was stored for further analysis. The samples were used 1 week after collection, to allow greater safety regarding infection of the trainer and the dogs to be trained. Dog training was performed at K9 International company which was largely experienced in dog training. Two healthy dogs, a male, and a female were used in this initial phase of the study. The dogs have been trained beforehand using operant conditioning (reward-based training) on an 8-station scent line. The tests were double-blind and performed in an isolated room without external stimuli. During the tests, only the dog and trainer were present in the room. Two metallic bars with four holders each were used to hold the samples, keeping a 50 cm space between each holder (Figure 2A). The sample was placed in the container by the experimenter, where one known PCR-positive sample was placed in a random holder and the other seven holders were kept with clean cotton balls as negative samples. After training was established, PCR-negative samples were used instead of clean cotton balls as negative samples to avoid confusing the dogs with smells of PCR false-negative samples. Sequentially, the trainer stimulated the dog to sniff every pot in the row, and when the dog sat in front of a sample (Figure 2B), the experimenter, a person from outside the room and following the test, indicated whether the dog was correct, and the trainer would praise and award the dog. Neither the trainer or the dogs knew which were the positive samples nor where they were being placed, therefore assuring the double-blind test. The sessions were video recorded. Five positive samples were used (1 in each daily test session) and each dog made about five daily attempts (walking along the line of supports and smelling the samples). The positive samples were placed at different spots in the line and the trials were independent of each other to assure randomized locations. The sensitivity for dogs to detect COVID-19 was calculated as the proportion of the number of correct indications of positive samples by the dogs, considering their joint and individual performance.

## Results

The results obtained showed that among the population sample (*n* = 100), 63% of volunteers were female and 37% male. In the analysis of the samples using the RT-PCR technique, 44% of the individuals tested positive for COVID-19. The mean age was 36.0 ± 2.1 among the positives, while for the negative ones, values of 40.2 ± 2.3 were obtained. Regarding the average body mass, 73.8 ± 2.4 kg was observed among the positives and 74.6 ± 1.8 kg among the negatives for COVID-19.

Concerning pre-existing diseases or comorbidities, only 30% of individuals who tested positive for COVID-19 reported any changes. However, among these, hypertension was reported in approximately 85% of cases in isolation (61.5%), associated with asthma (7.7%), or diabetes (15.4%). When asked about the main associated symptoms, headaches, loss of smell, fever, dry cough, and body aches were reported.

Considering individuals that tested positive for COVID-19, 63 (6%) were women and 36 (4%) were men. When comparing casuistry related to ethnicity, it was found that among the positive cases, the proportion between whites, blacks, and browns was 18.2, 38.6, and 43.2%, respectively. Regarding the experiments carried out with dogs, they correctly indicated 97.4% of the samples were positive for COVID-19. As for individual performance, the male animal showed 100% sensitivity while the female showed 95% sensitivity for the tests performed. Interestingly, they also consistently indicated one of the PCR-negative samples as positive, therefore, we decided to contact the patient and performed a new serological test, that took place 45 days after the first collection, where antibodies against SARS-COV2 were observed, confirming a false-negative result in the PCR, which was correctly identified by the dogs. Our tests showed that the dogs could predict the subject truly having the disease since the positive predictive value (PPV) was 100%, and reversely, the negative predictive value (NPV) when considering the probability of the subject not having the disease was 98.2% in accuracy.

## Discussion

Considering that current testing methods to screen SARS-COV2 in the population are costly, invasive, and time-consuming, in addition to the need to return to activities after quarantine and due to the expected endemicity of the disease in the human population, it has been crucial to find fast and safe screening methods which preferably, would not require individual sample collection and will allow the screening of a large number of individuals. In the perspective of One Health, considering the acquired knowledge among human, animal, and environmental scientists brought together, using dogs to detect the odor of COVID-19-positive patients has been shown to work favorably in at least four countries, such as France, Lebanon, Colombia, and Germany (14–16). One Health means that a balance is required to maintain life on Earth. With that in mind, we further explore the advantages of developing and applying this approach to detect COVID-19 using dogs. It impacts positively on the reduction of plastic and use of disposable materials, minimizes the environmental spread of the virus from undetected patients, reduces the invasiveness of current tests, and promotes early detection of infection. Moreover, since animals are still understudies for correctly identifying their role in the epidemiologic chain, dogs may become a sentinel and valid option for surveillance in other animals. The present study showed that trained dogs can detect COVID-19-positive patients based on the odor they release. They were also able to detect a PCR false-negative patient, as proved by serological testing of that same patient 45 days later. Our study reached 97.4% of success when considering both dogs. Similarly, other studies showed between 81 and 100% correct answers (14–16).

Regarding the type of sample, two studies (15, 16) used saliva or tracheobronchial secretions. Regardless, the dogs were still able to detect the odor, reaching similar results, despite samples being different from ours as we used a much safer sampler than infected respiratory samples. Another study (14) used the same type of sample presented in this study (axillary sweat), worked with eight dogs, and notably obtained about 83–100% success, with all of them significantly different from the percentage of success expected by chance alone. Whether other illnesses were associated with those patients was not the focus of this study, but future studies may show whether there is a connection between different diseases and detection.

Compared with other studies, the present study used less invasive clinical samples and presented a lower risk of infection for humans and contamination to the surrounding environment than those using oral and respiratory secretion samples (15, 16). Although the influence of the prevalence of diseases in the success of the testing needs to be accessed on field trials, this approach suggests that the dogs, given the opportunity, can access and screen patients without the hassle of taking any sample to a laboratory.

Considering the major disturbance caused by COVID-19 in everyday life, especially the necessity of returning the mobility of the population to a more regular level, the use of canine olfactive detection to identify COVID-19-positive individuals, a non-invasive technique using axillary sweat sample, has shown to become a very promising avenue. In this study, we have shown an above 97% success in identifying COVID-19-positive samples, including a false-negative RT-PCR sample by dog olfactive detection, with PPV of 100% and NPV above 98%. Taken together, these results and the statistics associated with them are extremely important to corroborate this successful One Health approach in Latin America as a means to reduce human, environmental, and animal risk of exposure to COVID-19.

## Data Availability Statement

The raw data supporting the conclusions of this article will be made available by the authors, without undue reservation.

## Ethics Statement

The studies involving human participants were reviewed and approved by Ethics Commitee of UFRPE: CEP UFRPE no. 4.112.622. The patients/participants provided their written informed consent to participate in this study. The animal study was reviewed and approved by Ethics Committee from UFRPE: CEUA UFRPE no. 6804230520.

## Author Contributions

AS, RM, LA, JS, FC, JP, VS, DG, and CJ in order to accomplish this work, from the intellectual process, every step involved in planning, designing the protocols, and analyzing the results and writing the article. All authors contributed to the article and approved the submitted version.

## Conflict of Interest

The authors declare that the research was conducted in the absence of any commercial or financial relationships that could be construed as a potential conflict of interest.

## Publisher's Note

All claims expressed in this article are solely those of the authors and do not necessarily represent those of their affiliated organizations, or those of the publisher, the editors and the reviewers. Any product that may be evaluated in this article, or claim that may be made by its manufacturer, is not guaranteed or endorsed by the publisher.

## References

[1] Roujian Lu XZ Li JP Niu P Yang B Wu H Wang W. Genomic characterisation and epidemiology of 2019 novel coronavirus: implications for virus origins and receptor binding. Lancet. (2020) 395:565–74. 10.1016/S0140-6736(20)30251-8 32007145 PMC7159086

[2] Editorial Comment. COVID19 and Globalization. One Health. (2020) 9:100132. 10.1016/j.onehlt.2020.100132 32368611 PMC7184197

[3] Sun J He WT Wang L Lai A Ji X Zhai X. COVID19: epidemiology, evolution, cross-disciplinary perspectives. Trends Mol Med. (2020) 26:483–95. 10.1016/j.molmed.2020.02.008 32359479 PMC7118693

[4] Leroy EM Gouilhb AR Brugere-Picouxc J. The risk of SARS-CoV-2 transmission to pets and other wild and domestic animals strongly mandates a one-health strategy to control the COVID19 pandemic. One Health. (2020) 10:100133. 10.1016/j.onehlt.2020.100133 32363229 PMC7194722

[5] Bai Y Yao L Wei T Tian F Jin DY Chen L. Presumed asymptomatic carrier transmission of COVID19. JAMA. (2020) 323:1406–7. 10.1001/jama.2020.2565 32083643 PMC7042844

[6] Goodell JW. COVID19 and finance: agendas for future research. Fin Res Lett. (2020) 35:101512. 10.1016/j.frl.2020.101512 32562472 PMC7152896

[7] Browne C Stafford K Fordham R. The use of scent-detection dogs. Irish Vet J. (2006) 59:97.

[8] Statheropoulos M Mikedi K Agapiou A Georgiadou A Karma S. Discriminant analysis of volatile organic compounds data related to a new location method of entrapped people in collapsed buildings of an earthquake. Anal Chim Acta. (2006) 566:207–16. 10.1016/j.aca.2006.03.023

[9] Rudnicka J Walczak M Kowalkowski T Jezierski T Buszewski B. Determination of volatile organic compounds as potential markers of lung cancer by gas chromatography–mass spectrometry versus trained dogs. Sens Actuat B Chem. (2014) 202:615–21. 10.1016/j.snb.2014.06.006

[10] Cornu JN Cancel-Tassin G Ondet V Girardet C Cussenot O. Olfactory detection of prostate cancer by dogs sniffing urine: a step forward in early diagnosis. Euro Urol. (2011) 59:197–201. 10.1016/j.eururo.2010.10.006 20970246

[11] Willis CM Britton LE Harris R Wallace J Guest CM. Volatile organic compounds as biomarkers of bladder cancer: sensitivity and specificity using trained sniffer dogs. Cancer Biomark. (2011) 8:145–53. 10.3233/CBM-2011-0208 22012770 PMC13015871

[12] Angle C Waggoner LP Ferrando A Haney P Passler T. Canine detection of the volatilome: a review of implications for pathogen and disease detection. Front Vet Sci. (2016) 3:47. 10.3389/fvets.2016.00047 27446935 PMC4919317

[13] Angle TC Passler T Waggoner PL Fischer TD Rogers B Galik PK. Real-time detection of a virus using detection dogs. Front Vet Sci. (2016) 2:79. 10.3389/fvets.2015.00079 26779494 PMC4705269

[14] Grandjean D Sarkis R Tourtier JP Julien C Desquilbet L. Detection dogs as a help in the detection of COVID19: can the dog alert on COVID19 positive persons by sniffing axillary sweat samples? Proof-of-concept study. PLoS ONE. (2020) 15:e0243122. 10.1371/journal.pone.0243122 33301539 PMC7728218

[15] Vesga O Valencia AF Mira A Ossa F Ocampo E Perez MA. Dog savior: immediate scent-detection of SARS-COV-2 by trained dogs. bioRxiv. (2020). 10.1101/2020.06.17.158105

[16] Jendrny P Schulz C Twele F Meller S von Köckritz-Blickwede M Osterhaus. Scent dog identification of samples from COVID19 patients–a pilot study. BMC Infect Dis. (2020) 20:1–7. 10.1186/s12879-020-05281-3 32703188 PMC7376324

[17] Barrios AW Sánchez-Quinteiro P Salazar I. Dog and mouse: toward a balanced view of the mammalian olfactory system. Front Neuroanat. (2014) 8:106. 10.3389/fnana.2014.00106 25309347 PMC4174761

[18] Craven BA Paterson EG Settles GS. The fluid dynamics of canine olfaction: unique nasal airflow patterns as an explanation of macrosmia. J R Soc Interface. (2010) 7:933–43. 10.1098/rsif.2009.0490 20007171 PMC2871809

